# Comparative Analysis of Prescriptions and Pharmacy Services in Internet-Based Psychiatric Hospital During and After the COVID-19 Pandemic: Retrospective Cross-Sectional Observational Study

**DOI:** 10.2196/74059

**Published:** 2026-03-04

**Authors:** Guowei Deng, Hui Xia, De-wei Shang, Yuguan Wen, Jinqing Hu, Yaqian Tan

**Affiliations:** 1 Department of Pharmacy, The Affiliated Brain Hospital, Guangzhou Medical University Guangzhou China; 2 Key Laboratory of Neurogenetics and Channelopathies of Guangdong Province and the Ministry of Education of China, Guangzhou Medical University Guangzhou China

**Keywords:** COVID-19, mental disorders, internet-based hospital, pharmacy services, prescriptions

## Abstract

**Background:**

The COVID-19 pandemic has significantly accelerated the development of internet-based hospitals and telepharmacy services. However, their characteristics and evolving trends remain unclear.

**Objective:**

This study aimed to assess the associations between distinct pandemic phases and the number of prescriptions, patients’ demographic characteristics, drug and disease distribution patterns, and pharmacy service indicators in our internet-based psychiatric hospital.

**Methods:**

In this retrospective cross-sectional observational study, we conducted a full-sample census of prescriptions issued in the internet-based psychiatric hospital of the Affiliated Brain Hospital of Guangzhou Medical University during November 2020-December 2023. Cancelled, pending, and test prescriptions were excluded, and no sampling procedure was used. The research timespan was divided into pandemic and postpandemic phases, and trends of prescriptions were evaluated using interrupted time series analysis. Outcome measures, including patients’ sex and age, diagnosed disease, drug type, pharmacist audit time, and audit outcome, were analyzed using the bootstrap method, Pearson chi-square analysis, and multinomial logistic regression.

**Results:**

The segmented regression model revealed significant positive correlation between months and number of prescriptions during pandemic phase (*F*_1,16_=6.96; *P*=.02), whereas no significant correlation was detected in postpandemic phase (*F*_1,10_=2.77; *P*=.13). Descriptive analysis with bootstrap method revealed that female population were the majority in the pandemic phase (7297/11,812, 61.78%; 95% CI 60.91%-62.70%) and the postpandemic phase (3520/5518, 63.79%; 95% CI 62.58%-65.18%). Young adults aged 18-40 years were the predominant population in the pandemic phase (5606/11,812, 47.46%; 95% CI 46.63%-48.39%) and postpandemic phase (2657/5518, 48.15%; 95% CI 46.79%-49.37%). Depressive disorder and quetiapine were the most frequently diagnosed disease and prescribed drug in both pandemic phases, respectively. The majority of prescriptions were audited within 5 minutes during the pandemic phase (5999/11,812, 50.79%; 95% CI 49.89%-51.65%), while most prescriptions were audited within 1-12 hours in the postpandemic phase (2031/5518, 36.81%; 95% CI 35.61%-37.95%). Pearson chi-square analysis and multinomial logistic regression indicated that variables positively correlated with pandemic phases included female (*P*=.01; odds ratio [OR] 1.09, 95% CI 1.02-1.17), aged ≤17 years (*P*<.001; OR 2.20, 95% CI 1.90-2.54), aged 18-40 years (*P*<.001; OR 1.59, 95% CI 1.38-1.83), audit time between 12 and 24 hours (*P*=.02; OR 6.26, 95% CI 1.38-28.49), and approved outcome (*P*=.03; OR 3.97, 95% CI 1.19-13.26). The audit time ≤5 minutes (*P*=.049; OR 0.22, 95% CI 0.05-0.99) was negatively correlated with the pandemic phases.

**Conclusions:**

This study innovatively applied descriptive and analytic statistical methods to evaluate the associations between different pandemic phases and the prescriptions and pharmacy services in an internet-based psychiatric hospital. This study addressed limitations of existing research through a larger sample size, longer research timespan, and analytic statistical methods. This study demonstrated early warning indicators and replicable analytic methods for other medical institutions and offered implications in optimizing the efficiency of pharmacy services.

## Introduction

### Background

The COVID-19 pandemic has profoundly affected the world and our daily lives [1-5]. In response to the COVID-19 outbreaks, the Chinese government had taken a range of actions, including temporary hospitals, lockdown, and quarantine [6,7]. However, these strict control measures inevitably disrupted conventional outpatient services, especially for patients with mental disorders [8]. Specifically, patients with chronic mental illness generally require long-term medication and regular medical assistance, and lockdown can cause difficulties in obtaining drugs and even the danger of discontinuation [9-11]. In addition, patients with long-term serious mental illness are often physically or socially disadvantaged, and COVID-19 highlighted these preexisting differences [12-15]. Finally, it has been reported that the global prevalence of mental health problems, such as loneliness, panic, anxiety, and depression, has increased significantly since the outbreak of the COVID-19 pandemic [16,17]. Hence, in order to meet the challenges of patients’ medical needs, and in line with the recommendation of the World Health Organization to strengthen the digitalization of health systems, online medical services have developed rapidly worldwide [18-20]. The National Health Commission of China has also promoted the establishment of internet-based hospitals and timely translated into emergency control measures of COVID-19 [21,22]. Internet-based hospitals in China have been reported to grow rapidly from approximately 1600 in June 2021 to over 3000 by June 2023 [23], demonstrating the growing importance of this online service model.

In fact, with the advancement of artificial intelligence, 5G networks, and virtual reality, internet-based hospitals have developed rapidly in the last decade [24]. Internet-based hospitals displayed significant advantages over offline services in terms of spatial accessibility and cost-effectiveness [25,26]. Further, internet-based hospitals established by physical hospitals can integrate online and offline medical information and offer consultation, prescription, and follow-up services to ensure the continuity of health care for patients [27]. It is worth mentioning that there has been a major problem of uneven distribution of medical resources in China, primarily manifested as great regional differences [28,29]. However, the establishment of internet-based hospitals has greatly improved the accessibility of high-quality medical resources in big cities, thereby narrowing the differences in medical resources in China [30,31].

Subsequently, with the outbreak of COVID-19, extensive evidence has emphasized the growing importance of telehealth in providing stable health care services and curbing the spread of the pandemic [32,33]. By transferring nonemergency cases to online services, internet-based hospitals significantly alleviated the overburden of health care systems, thereby optimizing the allocation of medical resources [34,35]. Additionally, pharmacy services, such as medication consultation, medication therapy management, patient education, and drug delivery services, have been shown to significantly reduce the financial burdens of patients and improve their medication adherence [25,36,37]. Despite these potential benefits, disparities among different populations might also be inadvertently exacerbated during the pandemic, including internet facility conditions, digital skills, and physical conditions [38-41]. Meanwhile, due to the disadvantages such as insufficient interaction between doctors and patients and poor timeliness, the quality of online health care services has received great concern [22,42,43].

As for patients with mental disorders, telepsychiatry digital platforms have fundamentally improved the accessibility of patients and exhibited therapeutic effects comparable to offline treatment [44,45]. To meet the growing medication needs of patients, our hospital (the Affiliated Brain Hospital of Guangzhou Medical University) launched an internet-based psychiatric hospital platform and officially started to provide online pharmacy services since November 2020. Specifically, patients can receive remote consultation from doctors at home, pay online, and settle in real time through medical insurance. Subsequently, electronic prescriptions are audited by trained and authorized pharmacists, and drugs are delivered directly to patients through qualified third-party logistics channels to reduce the spread of COVID-19.

### Relation to Previous Work

At present, most studies on the telemedicine services during the COVID-19 pandemic have focused on general hospitals [46-49], and few studies have explored the online pharmacy services in internet-based psychiatric hospitals [21,23]. Moreover, the limitations of existing studies in this area still exist, including small sample sizes, restricted study periods, and a lack of comparison between different pandemic phases. Besides, it is still unclear how the gradual resumption of normal medical services after the pandemic will affect internet-based hospitals. Thus, to fill these knowledge gaps, we conducted a retrospective cross-sectional observational study using the electronic prescriptions issued in our internet-based psychiatric hospital during November 2020 to December 2023. This study had a larger sample size and a longer research timespan than previous studies in this field and used analytic statistical methods to ensure the reliability of our findings. This study could gain in-depth insights into the development trends of prescriptions and key predictors of pharmacy services in internet-based psychiatric hospitals and offer empirical guidance for other medical institutions in the effective responses to public health emergencies.

### Objectives

In this study, we aimed to analyze the associations between different COVID-19 pandemic phases (predictor) and the trends and distribution patterns of the electronic prescriptions through descriptive and analytic statistical methods. The specific outcomes, including monthly prescription numbers, patients’ demographic characteristics (sex and age), clinical characteristics (primary diagnosed disease and type of drug), and pharmacy service indicators (pharmacist audit time and audit outcome), would be assessed to elucidate the changes in their distribution patterns driven by the pandemic phases.

## Methods

All methods and findings of this study were reported based on the STROBE (Strengthening the Reporting of Observational Studies in Epidemiology) guidelines [50] and the JARS (Journal Article Reporting Standards) guidelines [51,52]. A completed and filled out STROBE checklist of this study is provided as Multimedia Appendix 1.

### Study Design

This study was a retrospective cross-sectional observational study that comparatively analyzed the prescriptions and pharmacy services from an internet-based psychiatric hospital during November 2020 to December 2023. We used descriptive and analytic statistical methods to analyze the associations between the 2 distinct pandemic phases (pandemic phase and postpandemic phase) and the long-term trends of the prescriptions, patients’ demographic characteristics, drug and disease distribution patterns, and pharmacy service indicators.

### Settings and Data Collection

**Figure 1:**
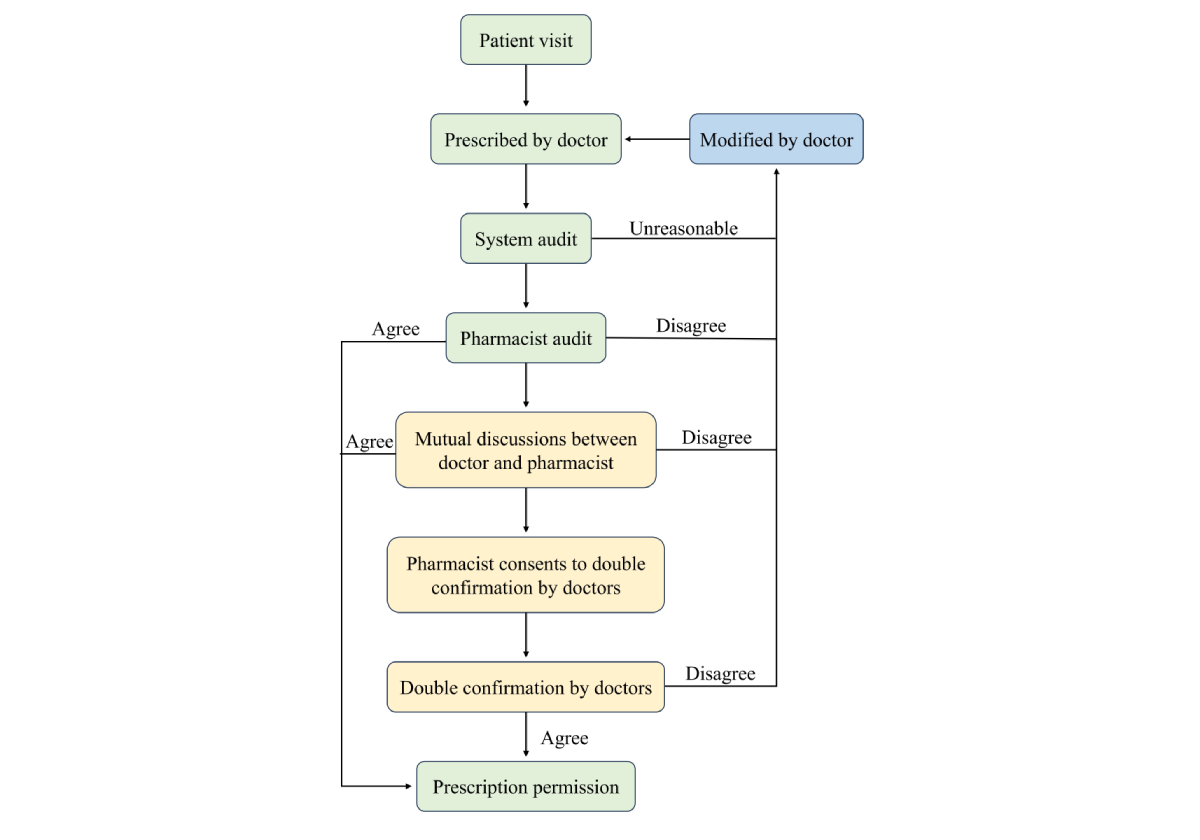
Flowchart depicting the doctor prescription and pharmacist audit process in the internet-based psychiatric hospital of the Affiliated Brain Hospital of Guangzhou Medical University during November 2020 to December 2023.

All data in this study were obtained from the internet-based psychiatric hospital of the Affiliated Brain Hospital of Guangzhou Medical University, a tertiary psychiatric hospital located in Guangzhou, China. The telepharmacy service platform was officially launched and started to provide online services since November 2020. Patients can visit our internet-based psychiatric hospital through the app developed by Guangdong Yunhui Technology Co, Ltd. After patients log on to the app and input their personal information, doctors will confirm their identity information and issue prescriptions according to patients’ condition. All electronic prescriptions from the internet-based psychiatric hospital are manually audited by trained and authorized pharmacists. The detailed workflow of the doctor prescription and the pharmacist audit process is demonstrated in Figure 1.

Data were collected from the electronic prescriptions of patients who received online medical services in our internet-based psychiatric hospital during November 1, 2020, to December 31, 2023. In this study, the exposure was the different pandemic phases. This study was a retrospective cross-sectional observational study, and no follow-up was performed. Data collection was conducted on a single day of March 11, 2024. Ultimately, a total of 17,330 electronic prescriptions with 36,088 drug records were identified and included in the final analysis.

### Eligibility Criteria

**Figure 2:**
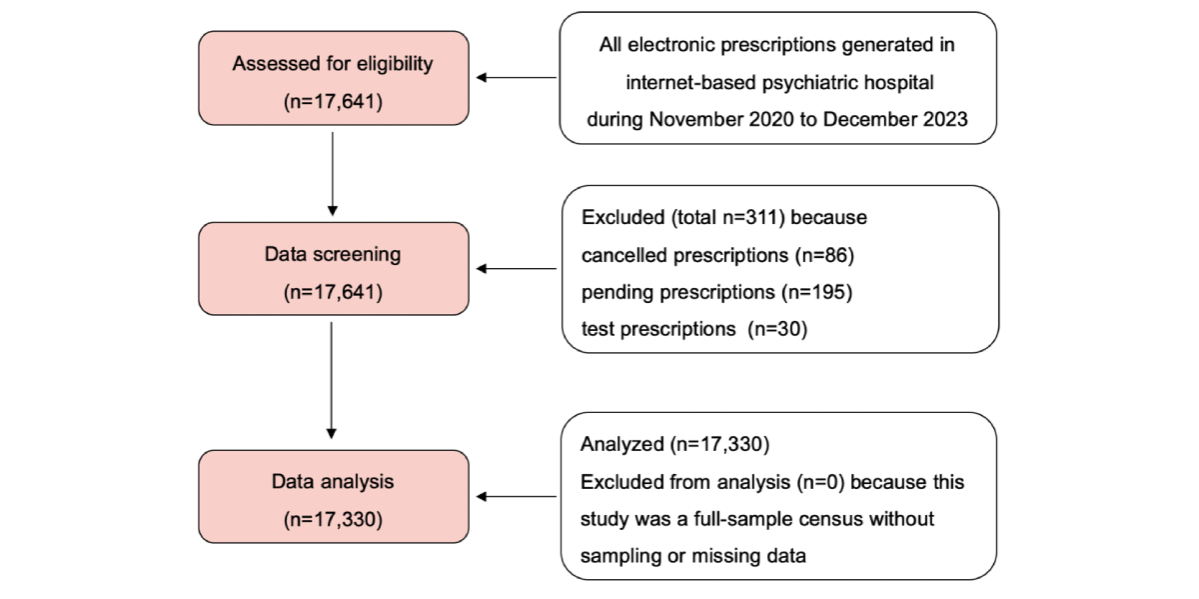
Flowchart demonstrating the eligibility criteria and data screening process of the electronic prescriptions issued in the internet-based psychiatric hospital of the Affiliated Brain Hospital of Guangzhou Medical University during November 2020 to December 2023.

This study aimed to analyze the entire population of prescriptions issued in our online platform during November 2020 to December 2023. There were no restrictions on prescription inclusion based on patients’ demographic characteristics (eg, sex, age, ethnicity, and socioeconomic status), and the inclusion criterion was defined as all prescriptions issued throughout the study period. Therefore, we essentially adopted a full-sample census approach. During the subsequent data screening process, cancelled, pending, and test prescriptions were considered to meet exclusion criteria and were removed from further data analysis. After data screening, a total of 17,330 prescriptions were included in the final data analysis. All data items of these included prescriptions were complete with no missing data. In addition, given the full-sample census method used, no sampling procedure was involved in this study. The detailed workflow of the eligibility criteria and data screening process is illustrated in Figure 2.

### Variables

In this study, the outcomes were measured at the prescription level, including the number of prescriptions, patients’ demographic characteristics (sex and age), clinical characteristics (primary diagnosed disease and type of drug), and pharmacy service indicators (pharmacist audit time and audit outcome). Within the standard framework of this observational research, the pandemic phases could theoretically be considered as potential “predictors” of the outcomes. The conditions in this study were all naturally observed, and no causal analysis of observed phenomena was performed. Thus, no variables were formally specified as confounders or effect modifiers.

### Bias

To prevent data inconsistencies due to periodic updates of the system when extracting data over multiple days, such as retrospective corrections of prescription status or batch updates of diagnoses and drug codes, all raw data in this study were extracted directly from our online platform backend system within a single day of March 11, 2024. This approach allowed us to ensure that all collected prescriptions had the same data status or version, thereby minimizing potential bias from data sources and guaranteeing data standardization.

### Data Sources or Measurement and Quantitative Variables

In December 2022, the Chinese government announced the end of all COVID-19 prevention and quarantine control measures in China. Based on this, the timeline of the COVID-19 pandemic was divided into 2 phases: the pandemic phase (November 2020-December 2022) and postpandemic phase (January 2023-December 2023). Exposure to the distinct COVID-19 pandemic phases was therefore defined and evaluated throughout the study period.

Based on the 2 pandemic phases divided, the number of monthly prescriptions and cumulative prescriptions were presented using a continuous time variable in months. Further, a 2-stage interrupted time series (ITS) analysis was conducted. In the pandemic phase, we adjusted the starting time point to July 2021 to exclude abnormal values (values deviating more than 2 times the CI from the absolute value) in June 2021. A segmented regression model in ITS analysis was used to evaluate the association between COVID-19 relaxation in China and the trends of monthly prescriptions [53,54]. A continuous time variable in months was applied in the model, and data were presented as absolute values with 95% CI. The *P* value <.05 was accepted as statistical significance in the segmented regression model.

Descriptive data of patients’ demographic characteristics, drug and disease distribution patterns, and pharmacy service indicators were presented using frequencies and percentages. The subgroups of age were assigned as follows: pediatric and adolescent (≤17 years), young adult (18-40 years), middle-aged (41-65 years), and older adult (≥66 years). The age of patients was assessed using median with IQR.

The pharmacy service indicators included pharmacist audit time and audit outcome. Pharmacist audit time was defined as the total length of time (in minutes) from prescription submission to audit completion, which was automatically recorded in our audit system. During data analysis, the audit time was divided into 7 subgroups according to our institutional pharmacy operation guidelines, including “≤5 minutes,” “5-30 minutes,” “30 minutes-1 hour,” “1-12 hours,” “12-24 hours,” “24-48 hours,” and “˃48 hours.” The audit outcomes mainly included “approved by pharmacists,” “double confirmation by doctors,” and “not approved by pharmacists.” We used consistent evaluation criteria for the audit outcomes of all prescriptions in this study according to our institutional pharmacy operation guidelines. To be specific, “approved by pharmacists” referred to prescriptions that meet the patient’s clinical condition and rationale for medication, “double confirmation by doctors” referred to prescriptions with potential medication errors (eg, inappropriate indication, repeated medication, and overdose) that required double confirmation by doctors, and “not approved by pharmacists” referred to prescriptions disapproved by pharmacists due to serious safety concerns (eg, contraindicated drugs and lethal doses).

To examine the robustness of the descriptive data, including patients’ sex and age, diagnosed disease, drug type, pharmacist audit time, and audit outcome, percentage values were sampled repeatedly for 1000 times using the bootstrap method and were presented as absolute values with 95% CI [55,56].

### Statistical Methods

Statistical data were presented for multiple variables, including sex, age, pharmacist audit time, and audit outcome. The general associations between pandemic phases and outcomes were examined using crosstabs analysis and Pearson chi-square analysis [54]. If the association was significant (*P*<.05), we further performed a multinomial logistic regression analysis to determine the source of significance using pandemic phases as a covariate [57]. Significance level of the Wald inclusion test statistic was applied with *P* value <.05. Odds ratios (ORs) with 95% CI and SE were calculated to quantify the associations between pandemic phases and outcomes. In this study, our predetermined α level was .05, and a 2-sided *P* value <.05 was considered statistically significant. All statistical analysis and graphical representations were performed using SPSS (version 26.0.0.2; IBM Corp) and GraphPad Prism (version 10.1.2; GraphPad Software, Inc).

### Ethical Considerations

This study was approved by the institutional review board (IRB) of the Affiliated Brain Hospital of Guangzhou Medical University following a thorough review of the research protocol (approval 2025111). The informed consent of this study was waived by the IRB, and the IRB allowed the primary data collection and secondary analysis of research data without additional consent. We confirm that this study adhered strictly to the principles of privacy and confidentiality protection. The research team complied with all relevant local, national, and international laws and regulations regarding the protection of personal information, privacy, and human rights. All sensitive data related to patients, including name, ethnicity, socioeconomic status, education level, residential address, contact information, geographic distribution, prescription cost, primary complaint, medical history, previous diagnosis, information of the clinician, and content of the conversation, were deidentified to ensure privacy and security. Besides sensitive information, all other collected data were reported in this study. This study involved prescription data from the local database of our hospital and did not involve human experimentation or compensation. We confirm that no personally identifiable information of patients was accessible to the research team. We confirm that no identification of individual participants or users in any images of the manuscript or supplementary material is possible.

## Results

### General Trends of Monthly Prescriptions

**Figure 3:**
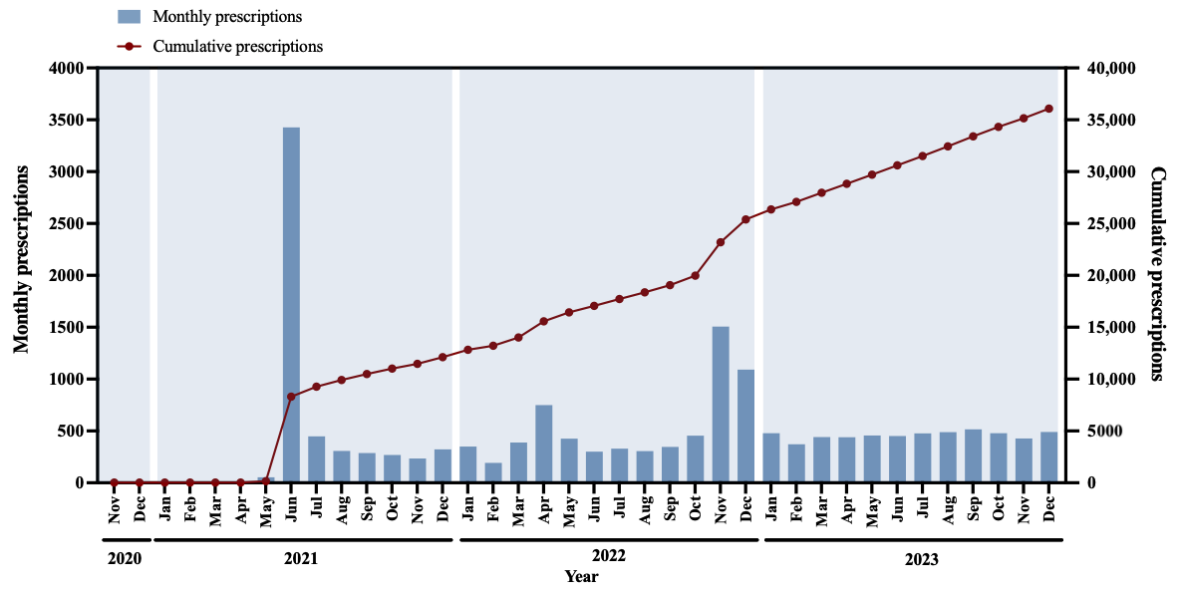
The number of monthly prescriptions and cumulative prescriptions in the internet-based psychiatric hospital of the Affiliated Brain Hospital of Guangzhou Medical University. Sources of data are all the electronic prescriptions issued during November 2020 to December 2023. Values are presented using a continuous time variable in months.

In this study, a total of 17,330 electronic prescriptions were finally identified, of which 11,812 prescriptions were processed during the pandemic phase, and 5518 prescriptions were issued during the postpandemic phase. As shown in Figure 3, the general trend of monthly prescriptions reflected a fluctuating tendency from November 2020 to December 2023. During the early stage of the pandemic phase from November 2020 to May 2021, the number of monthly prescriptions remained relatively low, with 76 prescriptions in total. Subsequently, the number of monthly prescriptions dramatically surged in June 2021 (n=3427), and then decreased to an average level of approximately 350 until another peak in November 2022 (n=1506). In the postpandemic phase, the number of monthly prescriptions stayed relatively stable around an average level of 460. On the other hand, the cumulative number of prescriptions exhibited a continuous upward tendency and peaked at 36,088 in December 2023.

### ITS Analysis of Monthly Prescriptions

**Figure 4:**
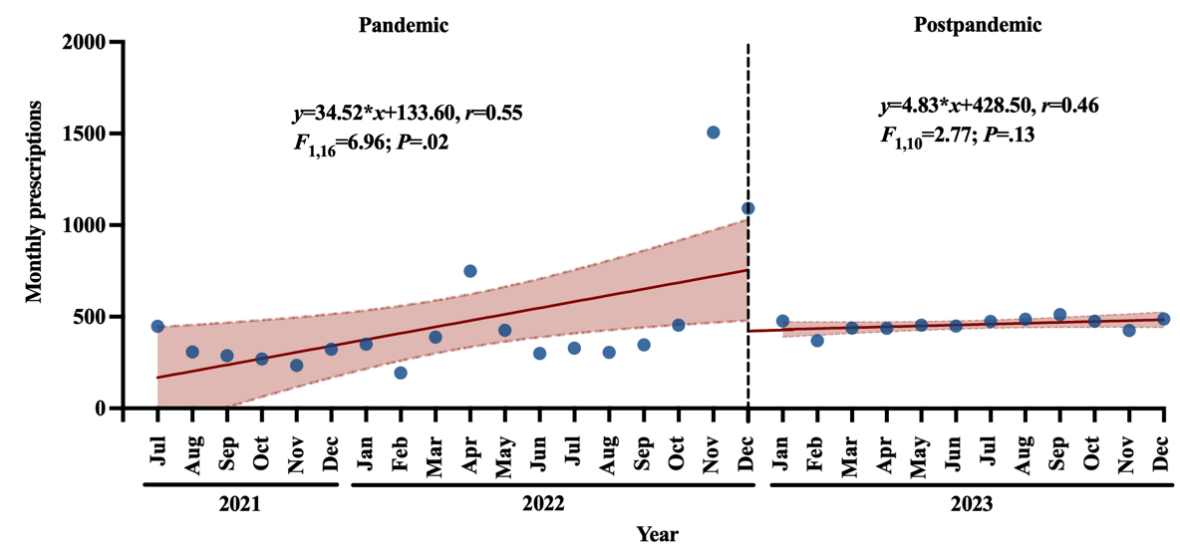
Segmented regression model in the 2-stage interrupted time series analysis evaluating the association between COVID-19 relaxation and the number of monthly prescriptions in the internet-based psychiatric hospital of the Affiliated Brain Hospital of Guangzhou Medical University. Sources of data are all the electronic prescriptions issued during July 2021 to December 2023. Values are presented as absolute values (blue dots) with 95% CI (red shadow) with a continuous time variable in months. The red solid lines indicate lines of regression, and the vertical black dashed line indicates the end of all COVID-19 prevention and quarantine control measures in China in December 2022. The *P* value <.05 indicates a significant difference between the regression line and the baseline.

A further ITS analysis was conducted to evaluate the association between COVID-19 relaxation and the number of monthly prescriptions. We conducted a 2-stage ITS analysis using the data ranging from July 2021 to December 2023. As depicted in Figure 4, the segmented regression model revealed a significant positive correlation between the months and the number of prescriptions (*y*=34.52*x+133.60; *r*=0.55; *F*_1,16_=6.96; *P*=.02; slope 34.52; 95% CI 6.78-62.27). These findings suggested an increasing tendency in patients’ needs and interest in our internet-based psychiatric hospital. In the postpandemic phase, the number of monthly prescriptions remained generally stable, and the trend was not statistically significant compared to baseline (*y*=4.83*x+428.50; *r*=0.46; *F*_1,10_=2.77; *P*=.13; slope 4.83; 95% CI –1.63 to 11.28). These findings indicated a stable and continuous service model of internet-based psychiatric hospital and a steady medication adherence of patients during January 2023 to December 2023.

### Demographic Characteristics of Patients

In this study, a total of 17,330 electronic prescriptions were collected, of which 11,812 prescriptions were issued during pandemic phase, and 5518 prescriptions were issued during the postpandemic phase. As the detailed characteristics of patients displayed in Table 1, the majority of patients were female during the pandemic phase (7297/11,812, 61.78%; 95% CI 60.91%-62.70%) and the postpandemic phase (3520/5518, 63.79%; 95% CI 62.58%-65.18%). There were 4515 male patients in the pandemic phase (4515/11,812, 38.22%; 95% CI 37.36%-39.03%) and 1998 male patients in the postpandemic phase (1998/5518, 36.21%; 95% CI 34.99%-37.32%).

**Table 1:** Demographic characteristics of patients who visited the internet-based psychiatric hospital of the Affiliated Brain Hospital of Guangzhou Medical University during November 2020 to December 2023^a^.

Characteristic		Pandemic (n=11,812), n (%)	95% CI	Postpandemic (n=5518), n (%)	95% CI	Chi-square (*df*)		*P* value	
**Sex**							6.5 (1)		.01
	Female	7297 (61.78)	60.91-62.70	3520 (63.79)	62.58-65.18				
	Male	4515 (38.22)	37.36-39.03	1998 (36.21)	34.99-37.32				
**Age (years)^b^**							295.4 (3)		<.001
	≤17	2873 (24.32)	23.47-25.19	1880 (34.07)	32.78-35.40				
	18-40	5606 (47.46)	46.63-48.39	2657 (48.15)	46.79-49.37				
	41-65	2400 (20.32)	19.61-21.07	703 (12.74)	11.92-13.59				
	≥66	933 (7.90)	7.42-8.36	278 (5.04)	4.48-5.62				

^a^Values are presented as absolute values and percentages with 95% CI using the bootstrap method. The general associations between different pandemic phases and sex and age of patients are evaluated with crosstabs analysis and presented with Pearson chi-square values.

^b^Pandemic: median 21 (IQR 18-44) years, postpandemic: median 21 (IQR 16-33) years.

Regarding age, young adults aged between 18 and 40 years accounted for the predominant population (5606/11,812, 47.46%; 95% CI 46.63%-48.39%) during pandemic phase, followed by patients aged ≤17 years (2873/11,812, 24.32%; 95% CI 23.47%-25.19%), patients aged between 41 and 65 years (2400/11,812, 20.32%; 95% CI 19.61%-21.07%), and patients aged ≥66 years (933/11,812, 7.90%; 95% CI 7.42%-8.36 %). Similarly, in postpandemic phase, young adults aged between 18 and 40 years accounted for the majority (2657/5518, 48.15%; 95% CI 46.79%-49.37%), followed by patients aged ≤17 years (1880/5518, 34.07%; 95% CI 32.78%-35.40%), patients aged between 41 and 65 years (703/5518, 12.74%; 95% CI 11.92%-13.59%), and patients aged ≥66 years (278/5518, 5.04%, 95 % CI 4.48%-5.62%). The median value of age was 21 (IQR 18-44) years during the pandemic phase and 21 (IQR 16-33) years in the postpandemic phase.

Overall, the Pearson chi-square analysis suggested significant statistical differences in sex distribution (*χ*^2^_1_=6.5; *P*=.01) and age distribution (*χ*^2^_3_=295.4; *P*<.001) between the 2 pandemic phases. These differences thereby suggested the necessity to perform further multinomial logistic regression analysis to determine the source of significance.

A follow-up multinomial logistic regression model was used to analyze the influences of different COVID-19 phases on the demographic characteristics of patients. As shown in Table 2, the postpandemic phase exhibited a positive correlation with the female group (*P*=.01; OR 1.09, 95% CI 1.02-1.17), suggesting a significantly increased proportion of female patients from 61.78% (7297/11,812) in the pandemic phase to 63.79% (3520/5518) in the postpandemic phase. Further, postpandemic phase displayed positive correlations with patients aged ≤17 years (*P*<.001; OR 2.20, 95% CI 1.90-2.54) and patients aged 18-40 years (*P*<.001; OR 1.59, 95% CI 1.38-1.83), suggesting significantly increased proportions of patients aged ≤17 years from 24.32% (2873/11,812) in pandemic phase to 34.07% (1880/5518) in postpandemic phase, and patients aged 18-40 years from 47.46% (5606/11,812) in pandemic phase to 48.15% (2657/5518) in postpandemic phase. We did not find a significant association between pandemic phases and patients aged ≥66 years across different pandemic phases (*P*=.83; OR 0.98, 95% CI 0.84-1.15).

**Table 2:** The influences of COVID-19 phases on the subgroups of patients’ demographic characteristics in a multinomial logistic regression model^a^.

Characteristic			SE		Wald		*df*		*P* value		OR^b^ (95% CI)
**Sex**											
	Female	0.03		6.51		1		.01		1.09 (1.02-1.17)	
	Male^c^	—^d^		—		—		—		1.00 (—)	
**Age (years)**											
	≤17	0.07		111.53		1		<.001		2.20 (1.90-2.54)	
	18-40	0.07		41.24		1		<.001		1.59 (1.38-1.83)	
	41-65	0.08		0.05		1		.83		0.98 (0.84-1.15)	
	≥66^c^	—		—		—		—		1.00 (—)	

^a^Data presented in this table are from the follow-up analysis of patients’ demographic characteristics to determine the source of the statistical difference. The SE and Wald inclusion test statistic are applied to quantify the associations between pandemic phases and outcomes.

^b^OR: odds ratio.

^c^Reference group.

^d^Not available.

### Distribution of Primary Diagnosed Diseases

As listed in Table 3, the top 10 primary diagnosed diseases in frequency were examined in our descriptive analysis. During the pandemic phase, a total of 83 diagnosed diseases were detected. The primary diagnosis of depressive disorder (3539/11,812, 29.96%; 95% CI 29.15%-30.79%) ranked first in frequency, followed by schizophrenia (2095/11,812, 17.74%; 95% CI 17.10%-18.40%) and bipolar disorder (1632/11,812, 13.82%; 95% CI 13.17%-14.50%). The primary diagnosis of epilepsy (108/11,812, 0.91%; 95% CI 0.77%-1.06%) ranked lowest among the top 10 primary diagnosed diseases. During the postpandemic phase, 58 diagnosed diseases in total were identified, and the leading 3 were depressive disorder (2094/5518, 37.95%; 95% CI 36.74%-39.12%), mood disorder (1191/5518, 21.58%; 95% CI 20.50%-22.74%), and schizophrenia (607/5518, 11%; 95% CI 10.20%-11.82%). In the postpandemic phase, epilepsy (49/5518, 0.89%; 95% CI 0.67%-1.12%) ranked lowest among the top 10 primary diagnosed diseases.

**Table 3:** The distribution of the top 10 primary diagnosed diseases from the electronic prescriptions processed in the internet-based psychiatric hospital of the Affiliated Brain Hospital of Guangzhou Medical University during November 2020 to December 2023^a^.

Rank		Primary diagnosed disease	Values, n (%)	95% CI
**Pandemic** (n=11,812)				
	1	Depressive disorder	3539 (29.96)	29.15-30.79
	2	Schizophrenia	2095 (17.74)	17.10-18.40
	3	Bipolar disorder	1632 (13.82)	13.17-14.50
	4	Mood disorder	1444 (12.22)	11.65-12.86
	5	Anxiety disorder	1208 (10.23)	9.61-10.82
	6	Obsessive-compulsive disorder	328 (2.78)	2.47-3.08
	7	Attention-deficit/hyperactivity disorder	232 (1.96)	1.73-2.22
	8	Alzheimer disease	164 (1.39)	1.19-1.61
	9	Autistic disorder	116 (0.98)	0.81-1.15
	10	Epilepsy	108 (0.91)	0.77-1.06
**Postpandemic** (n=5518)				
	1	Depressive disorder	2094 (37.95)	36.74-39.12
	2	Mood disorder	1191 (21.58)	20.50-22.74
	3	Schizophrenia	607 (11.00)	10.20-11.82
	4	Anxiety disorder	323 (5.85)	5.21-6.52
	5	Bipolar disorder	301 (5.45)	4.89-5.96
	6	Obsessive-compulsive disorder	162 (2.94)	2.56-3.35
	7	Attention-deficit/hyperactivity disorder	127 (2.30)	1.94-2.66
	8	Autistic disorder	117 (2.12)	1.81-2.45
	9	Alzheimer disease	72 (1.30)	1.04-1.59
	10	Epilepsy	49 (0.89)	0.67-1.12

^a^Values are presented as absolute values and percentages with 95% CI using the bootstrap method.

### Distribution of Drugs in Prescriptions

A total of 114 types of drugs prescribed in 25,402 times during the pandemic phase and 126 types of drugs prescribed in 10,686 times during the postpandemic phase were identified. As listed in Table 4, the top 10 drugs in frequency were examined in our descriptive analysis. During the pandemic phase, the predominant drugs in prescriptions were quetiapine (2653/25,402, 10.44%; 95% CI 10.03%-10.81%), lithium carbonate (1439/25,402, 5.66%; 95% CI 5.39%-5.93%), and escitalopram (1414/25,402, 5.57%; 95% CI 5.30%-5.85%). The drug agomelatine (870/25,402, 3.42%; 95% CI 3.20%-3.65%) had the lowest frequency among the top 10 prescribed drugs. Likewise, in the postpandemic phase, the top 3 most frequently prescribed drugs were quetiapine (1276/10,686, 11.94%; 95% CI 11.37%-12.55%), lithium carbonate (733/10,686, 6.86%; 95% CI 6.36%-7.37%), and escitalopram (710/10,686, 6.64%; 95% CI 6.20%-7.07%). Olanzapine (458/10,686, 4.29%; 95% CI 3.91%-4.71%) ranked lowest in the top 10 prescribed drugs during the postpandemic phase.

**Table 4:** The distribution of the top 10 drugs from the electronic prescriptions processed in the internet-based psychiatric hospital of the Affiliated Brain Hospital of Guangzhou Medical University during November 2020 to December 2023^a^.

Rank		Drug	Values, n (%)	95% CI
**Pandemic (n=25,402)^b^**				
	1	Quetiapine	2653 (10.44)	10.03-10.81
	2	Lithium carbonate	1439 (5.66)	5.39-5.93
	3	Escitalopram	1414 (5.57)	5.30-5.85
	4	Olanzapine	1398 (5.50)	5.26-5.75
	5	Sodium Valproate	1356 (5.34)	5.07-5.62
	6	Sertraline	1356 (5.34)	5.08-5.61
	7	Aripiprazole	1247 (4.91)	4.63-5.18
	8	Trihexyphenidyl	1185 (4.66)	4.41-4.93
	9	Tandospirone	932 (3.67)	3.46-3.89
	10	Agomelatine	870 (3.42)	3.20-3.65
**Postpandemic (n=10,686)^b^**				
	1	Quetiapine	1276 (11.94)	11.37-12.55
	2	Lithium carbonate	733 (6.86)	6.36-7.37
	3	Escitalopram	710 (6.64)	6.20-7.07
	4	Tandospirone	657 (6.15)	5.66-6.64
	5	Sodium valproate	612 (5.73)	5.31-6.14
	6	Sertraline	584 (5.47)	5.02-5.90
	7	Aripiprazole	570 (5.33)	4.90-5.75
	8	Lamotrigine	495 (4.63)	4.23-5.01
	9	Fluoxetine	460 (4.30)	3.93-4.67
	10	Olanzapine	458 (4.29)	3.91-4.71

^a^Values are presented as absolute values and percentages with 95% CI using the bootstrap method.

^b^Some prescriptions may contain more than 1 drug.

### Distribution of Prescription Audit Time and Audit Outcome

The detailed characteristics of prescription audit in the internet-based psychiatric hospital are listed in Table 5. During the pandemic phase, the majority of prescriptions (5999/11,812, 50.79%; 95% CI 49.89%-51.65%) were audited in ≤5 minutes, followed by prescriptions audited within 5-30 minutes (3255/11,812, 27.56%; 95% CI 26.74%-28.36%) and prescriptions audited within 1-12 hours (1466/11,812, 12.41%; 95% CI 11.86%-13.04%). The group of audit time ˃48 hours had the lowest number of prescriptions (4/11,812, 0.03%; 95% CI 0.01%-0.07%). In contrast, during postpandemic phase, most prescriptions (2031/5518, 36.81%; 95% CI 35.61%-37.95%) were audited within 1-12 hours, followed by prescriptions audited within 5-30 minutes (1359/5518, 24.63%; 95% CI 23.47%-25.73%) and prescriptions audited in ≤5 minutes (998/5518, 18.09%; 95% CI 17.13%-19.17%). Likewise, in the postpandemic phase, the group of audit time ˃48 hours had the lowest number of prescriptions (3/5518, 0.05%; 95% CI 0.01%-0.13%).

**Table 5:** The characteristics of prescription audit in the internet-based psychiatric hospital of the Affiliated Brain Hospital of Guangzhou Medical University during November 2020 to December 2023^a^.

Characteristic			Pandemic (n=11,812), n (%)		95% CI		Postpandemic (n=5518), n (%)		95% CI		Chi-square (*df*)		*P* value	
**Audit time**												2784.5 (6)		<.001
	≤5 minutes	5999 (50.79)		49.89-51.65		998 (18.09)		17.13-19.17						
	5-30 minutes	3255 (27.56)		26.74-28.36		1359 (24.63)		23.47-25.73						
	30 minutes-1 hour	957 (8.10)		7.65-8.57		669 (12.12)		11.31-13.01						
	1-12 hours	1466 (12.41)		11.86-13.04		2031 (36.81)		35.61-37.95						
	12-24 hours	86 (0.73)		0.57-0.88		404 (7.32)		6.64-8.07						
	24-48 hours	45 (0.38)		0.28-0.50		54 (0.98)		0.76-1.23						
	˃48 hours	4 (0.03)		0.01-0.07		3 (0.05)		0.01-0.13						
**Audit outcome**												601.0 (2)		<.001
	Approved by pharmacists	9844 (83.34)		82.68-84.06		5327 (96.54)		96.04-97.01						
	Double confirmation by doctors	1946 (16.47)		15.85-17.05		188 (3.41)		2.98-3.88						
	Not approved by pharmacists	22 (0.19)		0.12-0.25		3 (0.05)		0.01-0.13						

^a^Values are presented as absolute values and percentages with 95% CI using the bootstrap method. The general associations between different pandemic phases and audit time and audit outcome are evaluated with crosstabs analysis and presented with Pearson chi-square values.

As for audit outcomes, most prescriptions (9844/11,812, 83.34%; 95% CI 82.68%-84.06%) were approved by pharmacists during the pandemic phase. Of the 11,812 prescriptions in the pandemic phase, 1946 (16.47%; 95% CI 15.85%-17.05%) required double confirmation by doctors before approval, and 22 (0.19%; 95% CI 0.12%-0.25%) were not approved by pharmacists. Similarly, during the postpandemic phase, the vast majority of prescriptions (5327/5518, 96.54%; 95% CI 96.04%-97.01%) were approved by pharmacists. Of the 5518 prescriptions in the postpandemic phase, 188 (3.41%; 95% CI 2.98%-3.88%) required double confirmation by doctors before approval, and 3 (0.05%; 95% CI 0.01%-0.13%) were not approved by pharmacists.

Overall, the Pearson chi-square analysis revealed a significant statistical difference in audit time (*χ*^2^_6_=2784.5; *P*<.001) and audit outcomes (*χ*^2^_2_=601.0; *P*<.001) between the 2 pandemic phases. These differences thereby suggested the necessity to perform further multinomial logistic regression analysis to determine the source of significance.

A follow-up multinomial logistic regression model was used to analyze the influences of different COVID-19 phases on the characteristics of prescription audit. As shown in Table 6, the postpandemic phase exhibited a negative correlation with audit time ≤5 minutes (*P*=.049; OR 0.22, 95% CI 0.05-0.99), suggesting a significantly decreased proportion of prescriptions audited in ≤5 minutes from 50.79% (5999/11,812) in the pandemic phase to 18.09% (998/5518) in the postpandemic phase. There was a positive correlation with audit time within 12-24 hours (*P*=.02; OR 6.26, 95% CI 1.38-28.49), suggesting a significantly increased proportion of prescriptions audited within 12-24 hours from 0.73% (86/11,812) in the pandemic phase to 7.32% (404/5518) in the postpandemic phase. We did not find significant associations between different pandemic phases and audit time within 5-30 minutes (*P*=.44; OR 0.56, 95% CI 0.12-2.49), audit time within 30-60 minutes (*P*=.93; OR 0.93, 95% CI 0.21-4.17), audit time within 1-12 hours (*P*=.42; OR 1.85, 95% CI 0.41-8.27), or audit time within 24-48 hours (*P*=.55; OR 1.60, 95% CI 0.34-7.53).

**Table 6:** The influences of COVID-19 phases on the subgroups of prescription audit indicators in a multinomial logistic regression model^a^.

Characteristic			SE		Wald	*df*		*P* value		OR^b^ (95% CI)
**Audit time**										
	≤5 minutes	0.77		3.88		1	.049		0.22 (0.05-0.99)	
	5-30 minutes	0.76		0.59		1	.44		0.56 (0.12-2.49)	
	30 minutes-1 hour	0.76		0.01		1	.93		0.93 (0.21-4.17)	
	1-12 hours	0.77		0.64		1	.42		1.85 (0.41-8.27)	
	12-24 hours	0.77		5.63		1	.02		6.26 (1.38-28.49)	
	24-48 hours	0.79		0.35		1	.55		1.60 (0.34-7.53)	
	˃48 hours^c^	—^d^		—		—	—		1.00 (—)	
**Audit outcome**										
	Approved by pharmacists	0.62		5.01		1	.03		3.97 (1.19-13.26)	
	Double confirmation by doctors	0.62		0.31		1	.58		0.71 (0.21-2.39)	
	Not approved by pharmacists^c^	—		—		—	—		1.00 (—)	

^a^Data presented in this table are from the follow-up analysis of the indicators of prescription audit to determine the source of statistical difference. The SE and Wald inclusion test statistic are applied to quantify the associations between pandemic phases and outcomes.

^b^OR: odds ratio.

^c^Reference group.

^d^Not available.

Furthermore, the postpandemic phase displayed a positive correlation with the approved group (*P*=.03; OR 3.97, 95% CI 1.19-13.26), suggesting a significantly increased proportion of approved outcomes from 83.34% (9844/11,812) in the pandemic phase to 96.54% (5327/5518) in the postpandemic phase. We did not find significant associations between different pandemic phases and doctor double confirmation group (*P*=.58; OR 0.71, 95% CI 0.21-2.39).

## Discussion

### Principal Findings

In general, we found distinct trends of prescriptions in different phases of the pandemic, such that an overall upward trend during the pandemic phase and a stable tendency during the postpandemic phase. We found that female and young adults were the predominant groups, and their proportions increased with the development of the pandemic phases. Additionally, our findings demonstrated that the distribution patterns of primary diagnosed diseases and prescribed drugs were generally similar in both pandemic phases. At last, our results revealed that with the progress of the pandemic phases, the pharmacist audit time was extended, and the audit approval rate was increased.

### Detailed Discussion of the Findings

#### Interpretations

From the perspective of monthly prescriptions, we found that our internet-based psychiatric hospital exhibited distinct distribution patterns across 2 pandemic phases. Our results from ITS analysis might reveal a generally increasing need for patients in online pharmacy services during the pandemic phase and the ability of internet-based psychiatric hospitals to provide stable pharmacy services after the pandemic [58,59].

Initially, during November 2020-May 2021, the small number of cumulative prescriptions possibly indicated that patients have not yet adapted to the shift from offline to online medical treatment model [60]. Notably, in June 2021, with the major outbreak in Guangzhou, the outpatient services of our hospital were suspended, which led to a dramatic surge in the number of electronic prescriptions [61]. This finding was in line with previous evidence, indicating the public panic in the early stage of the pandemic and the increased demand for patients for internet-based hospitals [21,23]. Subsequently, from July 2021 to October 2022, with the optimization of human resources in our internet-based psychiatric hospital and the gradually reduced public panic [62,63], the average number of monthly prescriptions remained relatively stable at approximately 350. During November 2022-December 2022, with another major outbreak in Guangzhou, together with the subsequent COVID-19 control relaxation in China, the demand for internet-based psychiatric hospitals surged again [64]. In the postpandemic phase, the average number of monthly prescriptions was stable at approximately 460, which was consistent with previous findings, indicating the critical role of internet-based hospitals during and after the COVID-19 pandemic [65]. Importantly, our findings highlighted the capacity and necessity of internet-based psychiatric hospitals in providing long-term stable services after the COVID-19 pandemic [66].

Our results of prescription analysis indicated that female patients were the predominant population in our internet-based psychiatric hospital and were significantly associated with the COVID-19 pandemic phases compared to male patients. Previous studies have shown that female patients are more susceptible to external environmental stress compared to male patients, thereby resulting in mental health problems [67-70]. Recent evidence has suggested that female patients are more likely to seek help during public health crises, particularly during the COVID-19 pandemic [19,71]. Besides, our findings showed that patients aged 18-40 years were the major population in both pandemic phases, and individuals younger than 41 years of age were significantly associated with the pandemic phases. There were several driving factors that are worth noting. First, these individuals generally encounter significant life crises during the pandemic, such as academic disruptions, risk of unemployment, and financial instability [72]. Second, previous research has shown that the depression rate among adolescents and young adults has risen sharply over the past decade [73]. Fortunately, adolescents and young adults also demonstrated greater adaptability to digital health care platforms compared to the older population, which was helpful in facing public health crises [74,75]. Interestingly, our long-term datasets across pandemic phases appeared to reflect potential demographic differences based on sex and age, which echoed the previous evidence, indicating that the pandemic led to a widening digital divide [76]. Therefore, we suggest that future policy guidance and technical support should be committed to ensure balanced allocation of digital health care resources among different populations [77].

In this study, we found that the leading primary diagnosed diseases included depressive disorder, bipolar disorder, schizophrenia, mood disorder, and anxiety disorder. This disorder spectrum not only aligned with the clinical orientation of our internet-based psychiatric hospital but also suggested that the patient populations analyzed in this study met the intended service objectives of our online platform [23]. Previous studies have reported that sudden public health emergencies can cause various psychological disorders, such as depression, mood disorder, and anxiety [78,79]. During the COVID-19 pandemic, control measures of medical isolation or home quarantine were also reported to cause significant psychological pressure [80]. Our results revealed increased proportions of depressive disorder and mood disorder in the postpandemic phase, which might support the evidence that there is a pandemic-driven shift pattern in global mental health needs [81]. Regarding drug categories, quetiapine, lithium carbonate, and escitalopram were the top 3 drugs prescribed in both pandemic phases, which was consistent with the evidence that these drugs were the most commonly applied antipsychotics and antidepressants [82].

As for the time spent during the pharmacist audit process, it was reported that pharmacist audit time was significantly compressed during the pandemic due to strategic reallocation of hospital human resources [42,83]. In the postpandemic phase, we found a significantly decreased proportion of prescriptions audited in ≤5 minutes and a significantly increased proportion of prescriptions audited within 12-24 hours. Several factors could contribute to these changes. First, the increased complexity of prescriptions for patients infected with COVID-19 could lead to an extended audit time [23]. In addition, evidence has suggested that pharmacists were already at risk of burnout before the COVID-19 pandemic [84]. Subsequently, the increased workload coupled with decreased rest time have exacerbated the burnout, which might influence the overall prescription audit time [85].

Pharmacists are considered to play a crucial role in ensuring the quality of prescription audit [86]. Pharmacist audit offers greater flexibility and professional judgment than a system automatic audit by using their clinical experience in individual conditions of patients [23]. Our results suggested an overall high prescription approval rate in both pandemic phases, revealing the critical role of pharmacist audit in promoting rational medication use in the internet-based psychiatric hospital [87]. Additionally, our results indicated a significantly increased pharmacist approval rate as well as a decreased doctor double confirmation rate in the postpandemic phase. These findings possibly reflected the increased familiarity of pharmacists and doctors with the online platform and the technological optimization of the digital system in the postpandemic phase [88].

#### Innovation of the Study

To the best of our knowledge, this study is the first work that applied descriptive and analytic statistical methods to evaluate the associations between different phases of the COVID-19 pandemic and the prescriptions and pharmacy services in the internet-based psychiatric hospital. The analyzed indicators included long-term prescription trends, patients’ demographic characteristics, drug and disease distribution patterns, and pharmacy service indicators. Our results could provide practical experience for other medical institutions and help to promote the development of digital health service models in the future [89].

#### Comparisons to Existing Literature

In the field of pharmacy services in internet-based hospitals, most research has focused on the establishment and application of an artificial intelligence audit system [90,91], drug delivery [92-94], medication therapy management [95,96], and drug consultation [97,98]. To date, only a few studies have explored online pharmacy services related to the COVID-19 pandemic through prescription analysis. Among these studies, Chen et al [99] analyzed 1718 prescriptions from the online platform of a tertiary cancer hospital in China over 6 months, involving data of patients’ sex and age, geographical distribution, main diagnosis, and drug category. Ding et al [49] analyzed 1380 prescriptions from the online platform of a tertiary general hospital in China over 2 months, covering data of patients’ sex and age, residence, prescription department, payment of prescription, and drug delivery region. As for studies in the field of internet-based psychiatric hospitals, Xie et al [23] analyzed 2914 prescriptions over 24 months, and Du et al [21] analyzed 1259 prescriptions over 12 months*.* Notably, we found that the distribution patterns of patients’ sex and age, diagnosed diseases, and prescribed drugs in our study were generally consistent with these 2 studies [21,23], which supported the reliability and generalizability of our results.

However, existing studies conducting prescription analysis only applied descriptive statistical methods rather than analytic statistical methods to compare the results between different pandemic phases, limiting in-depth interpretation of the observed phenomena [21,23,49,99]. Our study addressed the limitations of existing research from several aspects. First, this study carried a larger sample size compared to previous studies, and the collection of 17,330 prescriptions strengthened the statistical power of the study [100]. Second, we provided a longer research timespan than previous studies, and the dataset over 38 months enhanced the robustness of our results [101]. Finally, by providing certainty of evidence through analytic statistical methods, including ITS analysis, bootstrap method, Pearson chi-square analysis, and multinomial logistic regression, we further explored the potential correlations between the pandemic phases and the observed outcomes [53-56].

#### Contribution of the Study

The main contribution of this study was to provide a fundamental practice model of telepharmacy service. First, we identified early warning indicators that can be used as key intervention steps in pharmacy services, such as extended audit time and an increased number of prescriptions [102]. Second, by using descriptive and analytic statistical approaches in the analysis of prescriptions and pharmacy services, we demonstrated replicable analytic methods that can be applied in other medical institutions, offering templates for future responses to public health crises [103]. Taken together, this study greatly enriched our understanding of pharmacy services in internet-based hospitals and provided practical experience for future multicenter research in this field [89].

#### Implications

Our study had direct real-world implications for optimizing the efficiency of pharmacy services in internet-based hospitals. Our data showed that prescription audit time was significantly extended in the postpandemic phase. Therefore, for hospital managers, it is recommended to establish a pharmacy personnel allocation system to timely deploy nonemergency personnel when prescription audit time increases abnormally [104]. In addition, it is important to build a reward mechanism for pharmacy services. Specifically, the efficiency of prescription audit can be promoted by implementing clear service standards and linking them to incentive funds [105]. For software developers, pharmacy service paths can be optimized for key populations. Our results suggested that the majority in internet-based psychiatric hospitals were female patients. Therefore, online psychological counseling tools can be developed for this population [106,107]. On the other hand, our results revealed the lowest proportion of older patients. Hence, in order to improve their user experience, simplified user interface and video chat tools can be added in the app [108]. Besides, we found that many prescriptions required double confirmation by doctors during the pandemic phase. To further improve doctor-pharmacist communication in this step, a real-time videoconferencing module can be added to the audit system [109,110]. Further, a local knowledge base can be built to predict prescriptions that may trigger double confirmation by doctors and convert them into pop-up notifications in the doctor-prescribing system [111].

### Limitations

This study had some limitations that need to be noted. First, although our study analyzed the real-world pharmacy service model in an operating online platform of a psychiatric hospital in China, its single-center nature might limit the generalizability of our findings in rural regions or other hospitals, such as general hospitals and rural primary medical institutions [112]. Future research of multicenter design should be required to further validate our conclusions [89]. Second, due to the observational nature of our study, the findings mainly allowed us to demonstrate statistical associations rather than definite proof of causality [113]. It would be helpful for future studies to manipulate more predictors in the model to clarify potential confounding effects [114]. Third, the absence of other unmeasured factors, such as prescription cost, patients’ geographic distribution, comorbidities, disease severity, and adverse events, might have influenced the depth of our findings [49,99]. The inclusion of these possible variables in future work would enrich our understanding of the internet-based hospital service models [115].

### Conclusions

In summary, this study applied descriptive and analytic statistical methods to evaluate the associations between different COVID-19 phases and the prescriptions and pharmacy services in our internet-based psychiatric hospital. We found an overall upward trend in the number of prescriptions during the pandemic phase and a steady trend after the pandemic. Among patients, female and young adults were the predominant groups, and their proportions increased with the development of pandemic phases. The distribution patterns of primary diagnosed diseases and prescribed drugs were generally similar in both pandemic phases. As the pandemic phases advanced, prescription audit time was extended, and the audit approval rate was increased. This study addressed the limitations of existing research by the application of larger sample size, longer research timespan, and analytic statistical methods. This study demonstrated early warning indicators and replicable analytic methods that can be applied in other medical institutions. Our findings also had implications for hospital managers and software developers in optimizing the efficiency of pharmacy services in internet-based hospitals.

## Data Availability

The datasets used and/or analyzed during this study are included in the paper or Multimedia Appendix 1; further inquiries are available from the corresponding author on reasonable request.

## Appendix

Multimedia Appendix 1 The completed and filled out STROBE checklist.

## Abbreviations

IRB: institutional review board
ITS: interrupted time series
JARS: Journal Article Reporting Standards
OR: odds ratio
STROBE: Strengthening the Reporting of Observational Studies in Epidemiology
